# Retraction: Influence of ATP-binding cassette transporters in root exudation of phytoalexins, signals, and in disease resistance

**DOI:** 10.3389/fpls.2013.00424

**Published:** 2013-10-23

**Authors:** Frontiers Plant Science Editorial Office

The Journal, Chief Editor and the Authors wish to retract the Original Research article cited above in its entirety. Based on information reported after publication, this article was found to have images that were inappropriately manipulated (Figure 1B: actin panel; Figure 6A: PR1, PR5; Figure 6B: AtATH6, AtATH10). The authors and the journal regret the errors and regret any inconvenience to the readers of Frontiers in Plant Science.

